# Nutrition Education for Emerging Adults: Protocol for Program Evaluation

**DOI:** 10.2196/81647

**Published:** 2026-01-15

**Authors:** Stephanie Rogus, Katelin Alfaro Hudak, Lilian O Ademu, Sumathi Venkatesh, Michael Laguros, Elizabeth F Racine

**Affiliations:** 1 Texas A&M AgriLife Research Center El Paso, TX United States; 2 Texas A&M AgriLife Extension Service College Station, TX United States

**Keywords:** emerging adults, nutrition education, curriculum development, program evaluation, extension

## Abstract

**Background:**

Emerging adults (ie, those aged 18 to 25 years) in the United States exhibit the poorest diet quality among all adult age groups, contributing to adverse health and academic outcomes. Existing nutrition education programs often overlook this population, particularly those without children.

**Objective:**

This study aims to present the development of a nutrition education curriculum for emerging adults, including its process and outcome evaluation strategies and measures.

**Methods:**

The Fuel to Thrive program was adapted from an established state program for adults. The content was informed by reviewing the literature, conducting focus groups with health educators, and holding regular meetings with a curriculum committee. The final program consists of five 1-hour lessons incorporating nutrition education, recipe demonstrations, and physical activity. Process evaluation will involve focus groups with participants and interviews with educators to assess relevance and feasibility. Outcome evaluation will include surveys administered before and after the program to assess dietary behaviors, physical activity, food safety, and food resource management.

**Results:**

The program is currently being piloted at a Texas university. Additional implementations are planned for fall 2025 and spring 2026, with the inclusion of the Short Healthy Eating Index Survey to better assess diet quality changes.

**Conclusions:**

The Fuel to Thrive program addresses a critical gap in nutrition education for emerging adults by offering a tailored curriculum. Future iterations will refine the program based on participant and educator feedback and expand evaluation efforts. The long-term goal is national dissemination to improve dietary behaviors and health outcomes among emerging adults.

**International Registered Report Identifier (IRRID):**

DERR1-10.2196/81647

## Introduction

Emerging adulthood (ages 18-25 years) is a pivotal developmental stage characterized by numerous life transitions, including entry into higher education or the workforce; increased financial and personal autonomy; and, for some, the assumption of parenting responsibilities [1,2]. This period also offers increased discretionary time and independence in decision-making, providing a critical window for establishing long-term health behaviors [1]. Despite its importance, this age group exhibits the poorest diet quality among all US adult populations, with average dietary patterns falling well below recommended nutritional guidelines [3].

Suboptimal diet quality among emerging adults has been associated with lower academic performance, poorer mental health outcomes, and low socioeconomic status [4-7]. These findings underscore the need to provide nutrition education and implement age-appropriate, evidence-based nutrition interventions for this population. Nutrition education programs that teach food preparation skills, food budgeting, and nutrition knowledge can potentially help emerging adults establish lifelong healthy eating behaviors [8-10]. Such programs offer practical, hands-on learning that builds confidence in preparing balanced meals, selecting nutritious options on a limited budget, and understanding the long-term health implications of dietary choices [11]. Beyond imparting knowledge, these interventions foster critical thinking about food systems, cooking methods, and the importance of meal planning, helping to combat common barriers such as time constraints, cost, and lack of culinary skills.

While existing state and federal programs focus on various aspects of diet-related behavior change, few are tailored specifically to emerging adults. For example, the Expanded Food and Nutrition Education Program (EFNEP), a US Department of Agriculture-funded Extension (outreach) program, serves families by delivering community-based nutrition education [12]. The program has historically focused on parenting populations, thereby overlooking the specific needs of emerging adults without children. The US Department of Agriculture only recently granted state EFNEP programs the ability to serve emerging adults, including those without children. In 2023, approximately 40 emerging adults without children enrolled in Texas EFNEP. A preliminary analysis of their data from the Web-Based Nutrition Education Evaluation and Reporting System [13] indicated an improvement in their Healthy Eating Index scores from a baseline of 43 to 51 after the intervention. While this suggests positive dietary change, the score still falls within the lowest category according to the National Cancer Institute’s recommended grading scale, reflecting poor overall diet quality [14]. Therefore, nutrition education programs that target specific needs and address persistent gaps in diet quality are warranted.

This paper presents the rationale, design, and proposed evaluation framework for a nutrition education intervention adapted from an existing EFNEP curriculum and tailored for emerging adults, that is, those aged 18 to 25 years. The program was developed as a 5-week, theory-informed intervention designed explicitly for this age group, regardless of educational or parental status. It retains EFNEP’s core emphasis on practical skill building in 4 domains—diet quality and physical activity, food resource management, food safety, and food security—while tailoring content to align with the life stage and challenges of emerging adults. The program aims to enhance nutrition knowledge, promote sustainable behavior change, and ultimately improve overall dietary health in this vulnerable age group.

## Methods

### Curriculum Development

Given the gap in nutrition education programs focused on emerging adults, the Fuel to Thrive (F2T) program was developed for an emerging adult audience, with initial testing to be conducted in a university setting. It was also designed for use within EFNEP programming. University health educators recognized that students would benefit from nutrition education that met their unique needs and noted that many students who participate in their programs receive federal financial assistance (eg, Pell Grants). During program development, the curriculum development team considered several pieces of information on emerging adults’ food choice and dietary habits, including (1) low diet quality and high prevalence of food insecurity; (2) newfound autonomy and key influences on food choice such as taste, convenience, and cost; (3) limited knowledge of basic nutrition, cooking, and food safety; and (4) low self-efficacy in food preparation. An Extension program specialist, a registered dietitian with experience in developing EFNEP programs, led the curriculum development, with input from other nutrition and health educators from the university and Extension, as described in the next paragraphs.

F2T is a modified version of Texas EFNEP’s “Healthy Bites, Healthy Moves” (HBHM) curriculum. The HBHM program is a series of 8 nutrition education lessons, informed by adult learning theory, covering healthy eating (using the MyPlate guide), family meal planning, grocery budgeting, food safety, and the importance of physical activity [15]. HBHM was created by Texas EFNEP in 2018, implemented in 2019, and revised in 2024. It was written to reflect the Dietary Guidelines for Americans and is currently offered only in Texas.

Before modifying the HBHM curriculum, a literature review on factors influencing emerging adult dietary behaviors was conducted, and 3 focus groups were held with EFNEP agents and educators familiar with educating emerging adults. Eleven staff (7 agents and 4 educators) from 8 counties across the state participated in the focus groups. Participants were asked to attend 1 of 3 informal virtual focus groups that lasted between 1 and 1.5 hours. Focus group participants reviewed the concepts and activities in the HBHM curriculum and discussed ways to tailor the program to meet the needs of emerging adults. These discussions helped identify topics important to emerging adults, areas in which additional education is needed, and strategies for relaying information in ways that emerging adults would retain and apply to their lives.

A curriculum review committee was formed in August 2024 and met weekly until mid-January 2025 to review and modify the HBHM curriculum for emerging adults. The committee included the curriculum developer (program specialist), 3 EFNEP staff members (the EFNEP coordinator and 2 EFNEP agents who had participated in the focus groups), and 2 staff members from the university’s health center. The curriculum was modified by reducing the number of lessons from 8 to 5. This was done, in part, because previous research suggests that short, focused curricula may improve program engagement, retention, and accessibility given the competing demands of emerging adults, including work, school, and social commitments [16-19]. The committee carefully considered each lesson and its content for relevance and redundancy to determine what could be removed without omitting important information. Examples of lessons that were removed include those focused on feeding children and the final summary lesson. Although EFNEP guidelines suggest that an 8-lesson program is standard [20], the number of lessons varies across programs [21-23], and EFNEP staff on the committee agreed that a 5-lesson series would be appropriate to deliver the core material while keeping emerging adults engaged throughout. Additionally, language and activities were adapted to focus on emerging adults rather than families, and campus-specific resources, such as nutrition facts information from campus dining options, were included. Other materials, such as Dietary Guidelines for Americans statistics and MyPlate resources, were updated, and information on protein and fueling performance was added. These modifications were supported by behavioral theory and nutrition education research, indicating that interventions tailored to developmental stage and lived experience are more effective in improving diet-related behavior [24,25].

The modified program, titled Fuel to Thrive, consists of 5 lessons, each approximately 1 hour in duration. This duration was deemed appropriate by the committee to adequately cover the material. Each lesson includes a PowerPoint (version 2511; Microsoft Corp) presentation, a recipe demonstration with tasting, and a physical activity component. The curriculum toolkit includes suggested enhancement items to help reinforce participant learning in each lesson (Table 1). Low-cost enhancement items that encourage program engagement are allowable expenses when offered as part of an EFNEP class [26].

**Table 1:** Fuel to Thrive program details.

Lesson number	Title	Lesson content	Enhancement items
Lesson 1	MyPlate, my goals	Goal setting Introduction to MyPlate, the Dietary Guidelines for Americans, and mobile nutrition apps Benefits of physical activity	Measuring cups
Lesson 2	Slice to serve	Fruit and vegetable groups (sources, nutrients, and benefits) Knife safety Knife skills, including slicing, dicing, chopping, and julienne (hands-on)	Cutting boards
Lesson 3	Read it to eat it	Dairy group (sources, nutrients, and benefits) How to effectively use a nutrition facts label Strategies to identify and decrease intake of saturated fats, sodium, and added sugars	Lacrosse balls
Lesson 4	Save money and eat better	Grains group (sources, nutrients, and benefits) Saving money at the store (budgeting, meal planning, shopping tips, and preparation tips)	Grocery list pad
Lesson 5	Protect your plate	Protein group (sources, nutrients, and benefits) Food safety (clean, separate, cook, and chill)	Food thermometer

### Evaluation Tool

#### Overview

The evaluation toolkit includes evaluations before and after the intervention. They are intended to be administered via Qualtrics (Qualtrics, LLC) at the beginning of the first lesson and the end of the final lesson. The presurvey evaluation contains 60 items, while the postsurvey evaluation includes 46 items. The surveys include questions from the Adult EFNEP Questionnaire assessing diet, physical activity, food safety, and food resource management, in addition to participant demographics. The Adult EFNEP survey was used because F2T is a modified EFNEP program with similar objectives. A description of the survey measures is outlined in the following sections.

#### Diet

The validated dietary behavior measures include several questions on consumption of targeted food groups and 1 question on the frequency of preparing meals at home [27]. The 10-item food frequency questionnaire asks participants about how many times per day (or per week) they consume certain foods or beverages, including vegetables, fruit, milk and soymilk, beans and peas, and regular soda. The food preparation question asks participants how many days a week they cook dinner (or their main meal) at home. Response options range from “I rarely cook dinner at home” to “6 to 7 days a week.”

#### Physical Activity

The Adult EFNEP Questionnaire includes several questions regarding participants’ physical activity levels. One question asks participants how many days in the past week they exercised for at least 30 minutes. The next question asks how many days they did strength-related workouts. A third asks how often they make small changes to be more active.

#### Food Safety

Four survey questions assess food safety practices. Participants indicate how often they wash their hands before preparing food, wash all items and surfaces that came into contact with raw meat or seafood, thaw frozen food at room temperature, and use a meat thermometer to check the internal temperature when cooking meat. Response options range from “never”=1 to “always”=5.

#### Food Resource Management

Nine validated survey questions assess food resource management practices [28]. These include how often participants compare food prices to save money, how often they plan meals before grocery shopping, and how often they use a written weekly or monthly food spending plan (refer to Multimedia Appendix 1 for the evaluation survey questions). Response options range from “never”=1 to “always”=5.

#### Demographic Variables

Questions are included to capture demographic characteristics, including year of birth, sex (male or female), race (American Indian or Alaska Native, Asian, Black or African American, Native Hawaiian or Pacific Islander, White, or other), Hispanic ethnicity (yes or no), occupation status (employed or self-employed full time, employed or self-employed part time, unemployed or unable to work, or student only), and current relationship status (single, in a relationship, married or in a domestic partnership, divorced or separated, or widowed). To gain some understanding of the environment in which participants live and resources available to them, additional questions ask about current living situation (campus or university housing, a family member’s home, or off-campus or other nonuniversity housing), whether they are on a university meal plan (yes or no), whether they are an international student (yes or no), and programs from which they receive assistance (Pell Grants, food assistance programs [eg, the Supplemental Nutrition Assistance Program], and other programs [eg, Temporary Assistance for Needy Families]).

### Curriculum Evaluation

The goal of the curriculum evaluation is to determine the program’s relevance, feasibility, and effectiveness. The specific objectives are (1) to assess the acceptability and relevance of the curriculum to emerging adults, (2) to determine the feasibility of implementing the program within a university setting, and (3) to examine the effectiveness of the program in improving participant knowledge and food-related behaviors.

The first 2 objectives are part of the process evaluation and will be addressed through focus group discussions with program participants and one-on-one, semistructured interviews with program educators. The third objective will be addressed by analyzing the quantitative data collected before and after the program and responses to behavioral change–related questions discussed in the focus groups.

#### Process Evaluation

The focus groups will be held in a separate room following the fifth lesson. A trained moderator will ask participants for their perspectives on the relevance, engagement, and usefulness of the course content and activities, along with questions to obtain feedback on improving the curriculum (refer to Multimedia Appendix 2 for the focus group questions). Video and audio of the focus groups will be recorded and later transcribed, and a member of the research team will be present to take notes. Focus groups will be conducted until data saturation.

Interviews with instructors will take place virtually 1 week after the final class. A trained interviewer will ask the instructors questions related to the overall flow and suitability of the curriculum, whether adjustments were made to the planned curriculum and activities, and barriers and facilitators to student recruitment and retention in the program (refer to Multimedia Appendix 3 for the interview questions). Interviews will be video- and audio-recorded and later transcribed.

The process evaluation will also include quantitative measures of the number of students who sign up for each class and the number of classes students attended. In the analysis, quantitative evaluation measures will be reported as means and SDs. The lead qualitative researcher will develop a codebook based on a priori descriptive categories, carefully read and code 1 interview and 1 focus group, and refine the codebook accordingly. Two other researchers will use this codebook to independently code 1 additional interview and 1 focus group, and then the 3 researchers will meet as a team to discuss discrepancies in coding decisions. The codebook will be revised based on the consensus reached during these discussions. Two researchers will then independently code the remaining interviews and focus groups using the updated codebook. Categories and supporting quotes will be compiled and presented.

#### Outcome Evaluation

Data collected before and after the program will be evaluated for program effectiveness. We will use a single-group, pretest-posttest design to assess outcomes before program initiation and at program completion. Two variables will be created for physical activity questions: (1) an indicator of whether participants meet current guidelines [29] (ie, engaging in any exercise on at least 5 days a week and resistance training on at least 2 days a week) and (2) how often the participants make small changes to be more physically active.

For diet behaviors, food safety, and food resource management, descriptive statistics will be used to summarize the presurvey and postsurvey responses to each question. For the main analysis, we will sum the responses to each question within a given category (ie, diet, physical activity, food safety, and food resource management) to form a scale in which higher numbers indicate greater use of each recommended practice or dietary behavior. This approach has been used in previous nutrition education research [30].

Participant demographic characteristics will be reported at baseline, and chi-square tests (categorical variables) and Wald tests (continuous variables) will be used to compare characteristics of those who completed the program with those who did not. The main analysis will be restricted to participants who completed the program and have both presurvey and postsurvey data. Outcome evaluation measures will be reported as proportions (categorical variables) or means and SDs (continuous variables) at baseline and at program completion. Generalized linear models with robust standard errors clustered at the participant level will be used to assess changes in outcomes from baseline (week 1) to program completion (week 5). Outcomes will also be examined by student participation in federal assistance programs to understand program effectiveness among low-income participants. The independent variable of interest will indicate whether the outcome was measured at baseline or after the intervention. Effect sizes and 95% CIs will be reported for each outcome. All quantitative analyses will be conducted using Stata (version 18.5; StataCorp LLC).

The focus group guide included a few outcome evaluation questions to assess participants’ perceptions of how the program shaped their (1) diet-related knowledge and skills, (2) self-efficacy to eat healthier, and (3) other health behaviors (ie, food resource management, food preparation, and food safety). Findings from the qualitative analysis of these questions will be used to offer a comprehensive understanding of the program’s outcomes.

### Ethical Considerations

The focus group guide and in-depth interview protocols, which include informing participants that they could opt out of the study and obtaining informed consent, have been reviewed and approved by the Texas A&M University Institutional Review Board (STUDY2025-0270). To ensure privacy, no identifiable information will be included in any write-up or presentation of the results. Participants will not receive compensation for participation. A separate institutional review board approval will be obtained for the analysis of deidentified secondary data collected in program surveys.

## Results

The F2T program is currently being pilot-tested by the university’s health center. In January 2025, the curriculum developer trained health center staff to deliver the curriculum. This involved providing the curriculum to the 4 educators who would teach the series a few weeks before a 2-day, in-person training. The educators then participated in virtual teach-backs 1 week later. In the teach-backs, the educators taught the class and were given time to ask questions, provide suggestions, and receive constructive feedback.

The health center advertised the program via social media, the health center’s online calendar of events, and a large sign displayed just inside the center. The 5 lessons were delivered in person at the health center over 4 weeks. Four educators were involved in program delivery, with one educator leading each lesson and another educator present to assist. Educators took turns leading the lessons so that each had the opportunity to lead at least 1 session.

Enhancement items were offered to all attendees at each lesson (Table 1), and participants were entered into a raffle to win 1 of 3 prizes, which were drawn and distributed at a meeting with participants following the fifth lesson. The 3 final prizes included an air fryer, a blender, and an electric skillet. Educators administered the evaluation tool described previously at the beginning and end of the program to measure program effectiveness.

The program will continue to be offered through fall 2025 and spring 2026. For these cohorts, we will include the Short Healthy Eating Index Survey to more accurately capture and quantify changes in participant diet quality [31]. This will allow comparison of effectiveness results with other program evaluations that examine diet quality while minimizing respondent and staff burden. We will also include select questions from the Young Adult Nutrition Literacy Tool to assess changes in confidence around healthy eating behaviors [32].

## Discussion

The transition to adulthood is characterized by newfound autonomy and responsibility, which may impact dietary behaviors and health. Despite having the lowest diet quality of all adults, emerging adults have been understudied and have not been targeted for nutrition interventions as a population distinct from general population adults. A nutrition education program developed specifically for emerging adults can fill this important nutrition intervention gap. In creating, implementing, and evaluating this program, we expect to find improvements in participants’ confidence around healthy eating behaviors and behaviors related to diet, food safety, physical activity, and food resource management at program completion. We also anticipate identifying areas for program improvement to better serve emerging adults and subsequently refining the program for future iterations.

The expected outcomes of the F2T program align with findings from nutrition education interventions that have been implemented among college student populations. Three such programs focused on nutrition education and cooking skills and were provided over 4 sessions, ranging from 15 minutes to 2 hours [33-35]. All 3 incorporated elements similar to those of the F2T program, including nutrition education lessons, cooking demonstrations, and tastings, along with other hands-on activities. The concepts covered, such as goal setting, cooking skills, healthy dietary patterns, and identifying healthy foods in the grocery store, also align with those covered in F2T; however, our program adds information on budgeting and physical activity. Each program reported positive impacts on self-efficacy related to cooking, fruit and vegetable consumption, or grocery shopping, or increased healthy food consumption following the program. Two interventions included follow-up assessments with participants either 4 or 6 months later and reported sustained increases in nutrition knowledge and healthy eating self-efficacy [33,35]. Together, these findings suggest that F2T may also be effective in improving confidence and dietary intake, and they underscore the importance of following up with participants in future research to better understand sustained changes and to revise the program as needed.

The strengths of this program and evaluation include modification of an existing EFNEP program using input from experts who have worked closely with emerging adult populations across multiple settings. We are also using an established EFNEP questionnaire in our evaluation, which will allow comparison of our results with those of other EFNEP programs. Additionally, the qualitative data we will collect will enable improvements to future program offerings and provide additional evidence on effectiveness. Limitations include students’ self-selection into the program, which likely reflects greater interest in nutrition and health compared with other emerging adults. The program will also include college students from 1 university, limiting the generalizability of the findings. Finally, there will be no comparison group in the evaluation.

We plan to disseminate our evaluation findings to academic audiences through presentations at research conferences and publications in peer-reviewed journals. We will also share our findings with Extension professionals through presentations at Extension-focused conferences and via a program brief. Future directions include considerations for program modifications (eg, additional sessions or engagement, different content, and tailored examples) based on the findings. We also plan to test the curriculum with emerging adult EFNEP participants without children in Texas. As part of this effort, we will consider the number of sessions needed for program graduation and compare outcomes from this program with those from longer EFNEP series. Additional efforts may include longer-term follow-up and the addition of a comparison group.

Our goal for this program is for it to be adopted by EFNEP and implemented at universities and in other settings with emerging adults across the United States following additional testing. We hope that this program will positively impact the dietary behaviors and health of emerging adults in the future.

## Appendix

Multimedia Appendix 1 Evaluation survey questions.

Multimedia Appendix 2 Focus group questions.

Multimedia Appendix 3 Interview questions.

## Abbreviations

EFNEP: Expanded Food and Nutrition Education Program
F2T: Fuel to Thrive
HBHM: Healthy Bites, Healthy Moves
