# Supplementary material for: Nutrition Education for Emerging Adults: Protocol for Program Evaluation
Source: JMIR Res Protoc. 2026 Jan 15;15:e81647. doi: 10.2196/81647 (PMC12856397; doi:10.2196/81647)
Supplement: Multimedia Appendix 1 [file resprot_v15i1e81647_app1.docx]

*Multimedia Appendix 1. Evaluation Survey.*

In what year were you born?

What was your sex assigned at birth?

- Female
- Male

Which race do you identify with?

- White or Caucasian
- Black or African American
- American Indian or Alaska Native
- Asian
- Native Hawaiian or Pacific Islander
- Other ______________________

Are you Hispanic or Latinx?

- Yes
- No

What is your current occupation status?

- Employed full-time/ self-employed
- Employed part-time
- Unemployed/ unable to work
- Student only

How would you describe your current relationship status?

- Single
- In a relationship
- Married or domestic partnership
- Divorced/ separated
- Widowed

What is your current living situation?

- Campus or university housing
- Parent/guardian/other family member's home
- Off-campus or other non-university housing

Are you on the university meal plan?

- Yes
- No

Are you an international student?

- Yes
- No

In the past 12 months, from which of the following programs did you receive assistance? (Select all that apply)

- Pell Grant
- Food assistance programs e.g., the Supplemental Nutrition Assistance Program (please specify) __________________________________________________
- Other support programs e.g., Temporary Assistance for Needy Families (please specify) __________________________________________________
- None of the above

Please mark the response that **best**describes how you **usually** do things.

How many **times a day** do you eat fruit? Examples of **fruits** are apples, bananas, oranges, grapes, raisins, melon and berries. Include fresh, frozen, dried, or canned fruit. *Do not include juice.*

- I rarely eat fruit
- Less than 1 time a day (a couple times a week)
- 1 time a day
- 2 times a day
- 4 or more times a day

How many **times a day** do you eat vegetables? Examples of **vegetables** are green salad, corn, green beans, carrots, potatoes, greens, and squash. Include fresh, canned, and frozen vegetables. *Do not count french fries, potato chips, or rice.*

- I rarely eat vegetables
- Less than 1 time a day (a couple times a week)
- 1 time a day
- 2 times a day
- 4 or more times a day

How many different kinds of vegetables do you usually eat a day?

- I rarely eat vegetables
- 1 kind a day
- 2 kinds a day
- 3 kinds a day
- 4 or more kinds a day

How many **times a day** do you drink milk or soymilk? *Do not count almond or coconut milk, or milk with cereal.*

- I do not drink milk
- I rarely drink milk
- 1 time a day
- 2 times a day
- 3 or more times a day

Over the last week, **how many days** did you eat red and orange vegetables? Examples of red or orange vegetables are tomatoes, red peppers, carrots, sweet potatoes, winter squash, and pumpkin.

- I did not eat red or orange vegetables
- 1 day a week
- 2 days a week
- 3 days a week
- 4 days a week
- 5 days a week
- 6-7 days a week

Over the last week, **how many days** did you eat dark green vegetables? Examples of dark green vegetables are broccoli, spinach, dark green lettuce, turnip greens, or mustard greens.

- I did not eat green vegetables
- 1 day a week
- 2 days a week
- 3 days a week
- 4 days a week
- 5 days a week
- 6-7 days a week

Over the last week, **how many days** did you eat beans and peas? Examples of beans and peas include pinto beans, black beans, navy beans, chili beans, refried beans, pork and beans, bean soup, barbeque beans, chickpeas, split peas, and black-eyed peas. Include beans from a can or cooked from dry.

- I did not eat beans and peas
- 1 day a week
- 2 days a week
- 3 days a week
- 4 days a week
- 5 days a week
- 6-7 days a week

Over the last week, **how many days** did you eat yogurt or drink smoothies with yogurt?

- I did not eat yogurt
- 1 day a week
- 2 days a week
- 3 days a week
- 4 days a week
- 5 days a week
- 6-7 days a week

Over the last week, **how many days** did you eat cereal with milk?

- I did not eat cereal with milk
- 1 day a week
- 2 days a week
- 3 days a week
- 4 days a week
- 5 days a week
- 6-7 days a week

How many **days a week** do you cook dinner (your main meal) at home?

- I rarely cook dinner at home
- 1 day a week
- 2 days a week
- 3 days a week
- 4 days a week
- 5 days a week
- 6-7 days a week

How often do you drink regular sodas (not diet)?

- Never
- 1-3 times a week
- 4-6 times a week
- 1 time a day
- 2 times a day
- 3 times a day
- 4 or more times a day

In the past week, **how many days** did you exercise for at least 30 minutes? This includes things like jogging, playing soccer, and doing fitness or dance classes, or exercise videos. This 30 minutes could be all at once or a few minutes at a time. *Do not count housework, taking care of your kids, or walking from place to place.*

- 0 days
- 1 day
- 2 days
- 3 days
- 4 days
- 5 days
- 6 days
- 7 days

In the past week, **how many days** did you do workouts to build and strengthen your muscles? This includes things like lifting weights and doing push-ups, sit-ups, or planks.

- 0 days
- 1 day
- 2 days
- 3 days
- 4 days
- 5 days
- 6 days
- 7 days

How often do you make small changes on purpose to be more active? This includes things like walking instead of driving, getting off the bus one stop early, doing a few minutes of exercise, or moving around instead of sitting while watching TV.

- Never
- Rarely (about 20% of the time)
- Sometimes (about 40% of the time)
- Often (about 60% of the time)
- Usually (about 80% of the time)
- Always

How often do you wash your hands with soap and running water before preparing food?

- Never
- Rarely (about 20% of the time)
- Sometimes (about 40% of the time)
- Often (about 60% of the time)
- Usually (about 80% of the time)
- Always

After cutting raw meat or seafood, how often do you wash all items and surfaces that came in contact with these foods?

- Never
- Rarely (about 20% of the time)
- Sometimes (about 40% of the time)
- Often (about 60% of the time)
- Usually (about 80% of the time)
- Always

How often do you thaw frozen food on the counter or in the sink at room temperature?

- Never
- Rarely (about 20% of the time)
- Sometimes (about 40% of the time)
- Often (about 60% of the time)
- Usually (about 80% of the time)
- Always

How often do you use a meat thermometer to see if meat is cooked to a safe temperature?

- Never
- Rarely (about 20% of the time)
- Sometimes (about 40% of the time)
- Often (about 60% of the time)
- Usually (about 80% of the time)
- Always

How often do you compare food prices to save money?

- Never
- Rarely (about 20% of the time)
- Sometimes (about 40% of the time)
- Often (about 60% of the time)
- Usually (about 80% of the time)
- Always

How often do you plan your meals before you shop for groceries?

- Never
- Rarely (about 20% of the time)
- Sometimes (about 40% of the time)
- Often (about 60% of the time)
- Usually (about 80% of the time)
- Always

How often do you look in the refrigerator or cupboard to see what you need before you go shopping?

- Never
- Rarely (about 20% of the time)
- Sometimes (about 40% of the time)
- Often (about 60% of the time)
- Usually (about 80% of the time)
- Always

How often do you make a list before going shopping?

- Never
- Rarely (about 20% of the time)
- Sometimes (about 40% of the time)
- Often (about 60% of the time)
- Usually (about 80% of the time)
- Always

How often do you use food coupons for food purchases?

- Never
- Rarely (about 20% of the time)
- Sometimes (about 40% of the time)
- Often (about 60% of the time)
- Usually (about 80% of the time)
- Always

How often do you use a written weekly or monthly food spending plan?

- Never
- Rarely (about 20% of the time)
- Sometimes (about 40% of the time)
- Often (about 60% of the time)
- Usually (about 80% of the time)
- Always

How often do you budget enough money for food purchases?

- Never
- Rarely (about 20% of the time)
- Sometimes (about 40% of the time)
- Often (about 60% of the time)
- Usually (about 80% of the time)
- Always

How often do you check for sales on foods **before** you shop?

- Never
- Rarely (about 20% of the time)
- Sometimes (about 40% of the time)
- Often (about 60% of the time)
- Usually (about 80% of the time)
- Always

How often do you check for food items on sale **when** you are at the store?

- Never
- Rarely (about 20% of the time)
- Sometimes (about 40% of the time)
- Often (about 60% of the time)
- Usually (about 80% of the time)
- Always

**The next section has statements people have made about their food situation. Choose the answer that best fits your food situation over the last 30 days.**

The food that I bought just didn’t last, and I didn’t have money to get more.

- Often true
- Sometimes true
- Never true
- Don't know

I couldn’t afford to eat balanced meals.

- Often true
- Sometimes true
- Never true
- Don't know

Did you ever cut the size of your meals or skip meals because there wasn’t enough money for food?

- Yes
- No
- Don't Know
