# Supplementary material for: Nutrition Education for Emerging Adults: Protocol for Program Evaluation
Source: JMIR Res Protoc. 2026 Jan 15;15:e81647. doi: 10.2196/81647 (PMC12856397; doi:10.2196/81647)
Supplement: Multimedia Appendix 2 [file resprot_v15i1e81647_app2.docx]

*Multimedia Appendix 2. Focus Group Questions*.

1. What do you think about your current eating habits?
2. If your eating habits could be healthier, what holds you back from eating healthy foods?
3. In what ways do your friends, family, and current living situation influence the way you eat?
4. What food preparation practices that you learned in the program have you been doing or plan to do?
5. How do you think the knowledge and skills you gained from the program will change the way you eat?
6. How confident do you feel in your ability to eat healthier after participating in the program?
7. What food safety practices that you learned in the program have you been doing or plan to do?
8. What are your thoughts on the usefulness of the lessons, activities, and handouts in each class and which ones stood out?
9. How would you describe your overall experience with the program?
10. What changes or additions to the program would you suggest?
11. Is there anything else you would like to share about your experience with the program?
