# Supplementary material for: Nutrition Education for Emerging Adults: Protocol for Program Evaluation
Source: JMIR Res Protoc. 2026 Jan 15;15:e81647. doi: 10.2196/81647 (PMC12856397; doi:10.2196/81647)
Supplement: Multimedia Appendix 3 [file resprot_v15i1e81647_app3.docx]

*Multimedia Appendix 3. Interview Questions*.

1. How did you find the overall flow of the curriculum for the target group?
2. How did you find the suitability of the curriculum for the target group? (*nudge: Were there any sections that felt particularly challenging to teach?)*
3. Were the lesson plans and materials easy to follow and implement? (*nudge: Can you provide examples of what worked well and/or what could be improved?*)
4. How did you feel about the amount of time allocated for each topic?
5. Were you able to follow the planned curriculum activities as intended? (*nudge: What adjustments did you have to make if not?*)
6. What do you think attracted the students to this program?
7. How did you recruit students to participate in the program? *(nudges: What barriers to recruiting students did you face, if any, and how did you overcome them? What barriers to retaining students did you face, if any, and how did you overcome them?*)
8. How engaged were the students during the classes? (*nudge: What activities or topics seemed to interest them most?*)
9. What feedback from participants stood out to you, and why?
10. How well did the training you received prepare you for delivering the curriculum? (*nudge: Were there any areas where you felt more support or training was needed?*)
11. What additional resources or training do you think would be beneficial for future implementations of this program?
12. How did you prepare to teach the curriculum?
13. If you were to teach this program again, what would you do differently?
14. Is there anything else you would like to share about your experience implementing the program that we haven’t covered?
